# Clinical and epidemiological profile of patients with valvular heart disease admitted to the emergency department

**DOI:** 10.1590/S1679-45082014AO3025

**Published:** 2014

**Authors:** Ricardo Casalino Sanches de Moraes, Marcelo Katz, Flávio Tarasoutchi

**Affiliations:** 1 Instituto do Coração, Faculdade de Medicina, Universidade de São Paulo, São Paulo, SP, Brazil; 2 Hospital Israelita Albert Einstein, São Paulo, SP, Brazil

**Keywords:** Heart valve diseases/epidemiology, Rheumatic fever/complications

## Abstract

**Objective:**

To evaluate the clinical and epidemiological profile of patients with valvular heart disease who arrived decompensated at the emergency department of a university hospital in Brazil.

**Methods:**

A descriptive analysis of clinical and echocardiographic data of 174 patients with severe valvular disease, who were clinically decompensated and went to the emergency department of a tertiary cardiology hospital, in the State of São Paulo, in 2009.

**Results:**

The mean age of participants was 56±17 years and 54% were female. The main cause of valve disease was rheumatic in 60%, followed by 15% of degenerative aortic disease and mitral valve prolapse in 13%. Mitral regurgitation (27.5%) was the most common isolated valve disease, followed by aortic stenosis (23%), aortic regurgitation (13%) and mitral stenosis (11%). In echocardiographic data, the mean left atrial diameter was 48±12mm, 38±12mm for the left ventricular systolic diameter, and 54±12mm for the diastolic diameter; the mean ejection fraction was 56±13%, and the mean pulmonary artery pressure was 53±16mmHg. Approximately half of patients (44%) presented atrial fibrillation, and over one third of them (37%) had already undergone another cardiac surgery.

**Conclusion:**

Despite increased comorbidities and age-dependent risk factors commonly described in patients with valvular heart disease, the clinical profile of patients arriving at the emergency department represented a cohort of rheumatic patients in more advanced stages of disease. These patients require priority care in high complexity specialized hospitals.

## RESUMO

**Objetivo:**

Avaliar o perfil clínico e epidemiológico dos pacientes com doença valvar que chegaram descompensados no pronto atendimento de um hospital universitário de referência nacional.

**Métodos:**

Análise descritiva de dados clínicos e ecocardiográficos de 174 pacientes consecutivos portadores de doença valvar grave, que apresentaram descompensação clínica e procuraram atendimento no pronto-socorro de um hospital terciário de cardiologia do Estado de São Paulo no ano de 2009.

**Resultados:**

Dos 174 pacientes avaliados, a média etária foi de 56±17 anos e 54% eram do gênero feminino. A principal etiologia da doença valvar foi a reumática (60%), seguida pela doença degenerativa aórtica (15%) e pelo prolapso mitral (13%). A valvopatia mais comumente observada de forma isolada foi a insuficiência mitral (27,5%), seguida por estenose aórtica (23%), insuficiência aórtica (13%) e estenose mitral (11%). Nos dados ecocardiográficos, a média do diâmetro do átrio esquerdo foi de 48±12mm, do ventrículo esquerdo na sístole foi de 38±12mm, e diástole foi de 54±12mm; a média da fração de ejeção foi de 56±13% e a pressão pulmonar média foi de 53±16mmHg. Aproximadamente metade dos pacientes (44%) estava em fibrilação atrial, e mais de um terço dos pacientes (37%) já havia sido submetido a outra cirurgia cardíaca.

**Conclusão:**

Apesar do aumento das comorbidades e dos fatores de risco idade dependentes comumente descritos nos portadores de cardiopatia valvar, o perfil clínico dos sujeitos que chegaram ao pronto atendimento representou uma coorte de pacientes reumáticos em estágios mais avançados de doença. Esses pacientes requerem atendimento prioritário em serviço especializado de alta complexidade.

## INTRODUCTION

The rheumatic etiology prevails as the main cause of valvular heart disease in Brazil,^(1)^ and it is quite different from Europe and the United States, where the disease is mainly caused by degenerative conditions.^(2)^

The population is aging in Brazil. Life expectancy at birth, according to data from the Information Technology Department of the Unified Health System (DATASUS), is roughly 73 years^(3)^ and this aging has increased the diagnosis of degenerative etiology valve diseases, frequently accompanied by age-dependent comorbidities.

This kind of patient is a real challenge to the general practitioner at the front line of care, given comorbidities plus underlying valve disease make treatment complex, and clinical outcomes are not always satisfactory.

In view of this increasing complexity, specialists in valve disease have proposed at cardiology associations a multidisciplinary professional team (the heart team) approach for these patients, including cardiologists, chest physicians, geriatrists, heart surgeons, dietitians, radiologists, hemodynamic specialists and psychologists, among others.

University hospitals are essential parts of the Brazilian health system, because they are references for highly complex cases in the entire national territory. However, due to the difficulties in access found in our health system, patients who should receive preventive care at the initial stages of valve heart disease at primary care units eventually reach tertiary and quaternary hospitals at advanced stages of the valve heart condition, requiring urgent intervention through the emergency department.

The clinical characteristics and comorbidities of patients with valve heart disease who arrive decompensated at the emergency department have not been acknowledged. Therefore, it is essential for the general practitioner to acknowledge the epidemiological, clinical and echocardiographic profile of individuals with valve disease who arrive at the emergency department.

## OBJECTIVE

To evaluate the epidemiological, clinical and echocardiographic profile of patients with valvular heart disease who arrive decompensated at the emergency department of a national reference university hospital.

## METHODS

A descriptive analysis of clinical and echocardiographic data of 174 consecutive patients with severe valve disease, who arrived clinically decompensated at the emergency department, and were admitted to a tertiary cardiology hospital in the State of São Paulo, in 2009.

All patients included had valve heart disease as their main diagnosis and cause of their clinical instability. Regardless of the type of valve disease, it was confirmed by the clinical valve disease unit team, and through echocardiographic assessment. All patients were functional class III/IV of the New York Heart Association.

The clinical profile variables collected upon admission were:

– Age
– Gender
– Underlying valve disease
– Etiology of valve disease (based on clinical history, physical exam and echocardiography)
– Comorbidities: hypertension defined as blood pressure >140×90mmHg or use of antihypertensive drugs; *diabetes mellitus* defined as need for oral hypoglycemic agents and/or insulin; coronary artery disease; peripheral artery disease or previous neurological disorder
– Previous heart surgery
– Presence of valve prosthesis
– Atrial fibrillation (paroxistical, persistent and permanent)
– Medication taken by patient

The echocardiogram after admission registered: left atrial and both ventricular diameters; pulmonary artery systolic pressure (PASP); diastolic function (ventricular filling pattern); left ventricular ejection fraction (LVEF); estimate of right ventricular function; and analysis of valves. PASP was classified into two groups: high pressure, if PASP >30mmHg, and normal, if PASP <30mmHg. LVEF >50% was considered normal and LVEF <50%, decreased.

Continuous variables were expressed as mean and standard deviation (normal distribution), and median and interquartile variation (asymmetrical distribution). Categorical variables were expressed as absolute and relative frequencies. The study protocol was approved by the Research Project Analysis Ethics Committee (Cappesq) on April 7, 2010, with protocol number 0155/10.

## RESULTS

Among the 174 patients evaluated, the mean age was 56±17 years and 54% were female. The main valve etiology was rheumatic in 60%, followed by degenerative disease in 15% of patients (Table 1). The most frequently observed isolated valve disease was mitral regurgitation (27.5%), followed by aortic stenosis (23%). In relation to comorbidities, 51% had hypertension, 16% *diabetes mellitus* and 44% atrial fibrillation. Over one third of patients had already been submitted to another heart valve surgery and 95% had a biological prosthesis. Valve prosthesis dysfunction was the reason for admission for 9.2%; the main prosthesis was mitral, in 8.1%, followed by the aortic. Associated coronary artery disease was found in 17% of patients (Table 1).

**Table 1:** Patients' characteristics

Clinical findings		Mean and/or %
Age, years		56±17
Female		54
Hypertension		51
*Diabetes mellitus*		16
Atrial fibrillation		44
Reoperations		37
Valve disease		
	Mitral regurgitation	27,5
	Aortic stenosis	23
	Aortic regurgitation	13
	Mitral stenosis	11
Etiology		
	Rheumatic	60
	Aortic degenerative disease	15
	Mitral valve prolapse	13
	Endocarditis	9
	Others*	3

* Bicuspid aortic disease, ischemic disease and aneurysm and aorta dissection.

Regarding medication use, 80% were on diuretics, 55% on angiotensin-converting enzyme inhibitors, 33% on aldosterone antagonists, 44% on digoxin, 35% on beta blockers, 17% on aspirin, 25% on anticoagulants and 10% on amiodarone (Table 2).

**Table 2:** Medication used

Medication	Use (%)
Diuretics	80
Angiotensin-converting enzyme inhibitors	55
Angiotensin II receptor inhibitors	12
Calcium antagonists*	13
Digoxin	44
Beta blockers**	35
Aldosterone antagonist	33
Aspirin	17
Anticoagulants	25
Statins	27
Amiodarone	10
Hydralazine/nitrate	12

* The main calcium antagonist taken was amlodipine;

** the main beta blocker taken was carvedilol.

As to echocardiographic parameters (Table 3), the mean left ventricle dimension on systole was 38±12mm, and on diastole, 54±12mm. Mean LVEF was 56±13%. LVEF was classified as normal (LVEF>50%) in 77%, and decreased (LVEF<50%) in 23%. Mean PASP was 53±6mmHg, in that 55% of patients had a high pulmonary pressure (PASP>30mmHg) and 45% had a PASP<30mmHg. All patients were in functional class III or IV upon admission at the emergency department and the main reason for clinical decompensation was progression of the uderlying disease. Most patients had already been assessed by specialists at the outpatient clinic, and over 90% of them were already programmed for surgery due to progression of symptoms and echocardiography parameters.

**Table 3:** Echocardiographic parameters

Echocardiography	Values
Left atrium, mm	48±12
LVSD, mm	38±12
LVDD, mm	54±12
LVEF, %	56±13
PASP, mmHg	53±16

LVSD: left ventricular systolic diameter LVDD: left ventricular diastolic diameter; LVEF: left ventricular ejection fraction; PASP: pulmonary artery systolic pressure.

## DISCUSSION

The main findings of the present study were: most patients with valve disease seen at the emergency department had rheumatic etiology; despite the main rheumatic etiology, the mean age of patients was above what is expected for this etiology; a significant part of patients seen had already been submitted to heart surgery; most patients seen were already scheduled for surgery.

In rheumatic disease, the eradication of the infectious condition of the oropharynx would abort the emergence of immune disease and its complications.^(4)^ However, in Brazil, primary care and access to health services are far from ideal, and rheumatic fever still has a high incidence. In our study, in fact, the rheumatic etiology was the most frequent. In general, rheumatic patients are affected at an earlier age (school and adolescence) and, depending on the degree of valve involvement, consequences can appear at very early ages. There is predominance in female patients and the mitral valve is the most affected.

In our study, most patients were women and mitral involvement was very common. However, the mean age of patients was above expected (considering rheumatic etiology). We highlight that patients had more advanced stage valve disease characterized by previous valve surgery and the frequent presence of atrial fibrillation and pulmonary hypertension.

In the natural history of valve heart disease, the presence of valve disease-related symptoms is the main indication for repair. However, some echocardiographic parameters, such as ventricular function, pulmonary artery pressure and ventricular diameters, are also taken into account to indicate intervention.^(1)^ Particularly, atrial fibrillation and pulmonary hypertension also have prognostic meaning. Pulmonary artery pressure is related to an increased risk of death and heart failure.^(5)^

Atrial fibrillation, which is responsible for reducing cardiac output and predisposes to thromboembolic events,^(6)^ also is an independent predictor of risk of death in the postoperative period of heart surgery, as exposed in the STS risk score.^(7)^

Another striking characteristic of this population was the number of previous surgeries that, in this case, can be explained by the rheumatic etiology, in which the first surgery occurred at a young age, with the preference of the organization for biological prostheses. This choice is guided by two main reasons: the chronic use of anticoagulants would make pregnancy difficult in women at child-bearing age given the medication is mandatory with a mechanical prosthesis, unlike bioprostheses; second, the difficult access to medical care and the low level of schooling would be inversely related to appropriate compliance to and control of oral anticoagulants.^(8)^

Regarding surgery in youth, we know that the durability of a biological prosthesis can be of up to 15 to 20 years in the best scenarios, but its dysfunction may happen at any moment during follow-up. This fact would be one more factor to explain the high incidence of reoperations.

The mean ventricular function was not as low as expected in a population with important symptoms (functional classes III/IV), but many cases were of individuals with single or combined mitral regurgitation, which knowingly overestimates ventricular function.

Most patients had already been assessed by a specialist in the outpatient clinic and over 90% of them were already scheduled for surgery. However, due to the imbalance between demand and availability for intervention in a programmed surgical procedure, in many cases patients were on hold for treatment, and were followed by clinical outpatient care. During this period, many patients were eventually admitted to hospital in unfavorable conditions and at advanced stages of poor heart adaptation. Non-programmed surgery, as an emergency, is a reality in our country due to high demand, poor distribution and poor organization of the healthcare system. This delay has already been described in other centers in the world.^(9)^

Surgery triggered through the emergency department is an independent variable for operative risk and, in the main heart surgery risk scores, such as the EuroSCORE, it represents an independent risk variable.^(10)^

## LIMITATION

The present study is descriptive of one center and limited in time. There are no inferential possibilities. However, it adds relevant information on the “real world” given it was performed at a high complex valve heart disease reference center.

## CONCLUSION

Despite increase in comorbidities and age-dependent risk factors commonly described in individuals with valvular heart disease, the clinical profile of patients who present at the emergency department represented a typical cohort of rheumatic patients at more advanced stages of the disease. Mitral disease, young patients (in comparison to international valve disease cohorts), high rate of reoperations and pulmonary hypertension prevailed. These patients require priority care at high complexity specialized services.
